# Gaze Following as an Early Diagnostic Marker of Autism in a New Word Learning Task in Toddlers

**DOI:** 10.1007/s10803-023-06043-1

**Published:** 2023-07-06

**Authors:** Raquel Camero, Carlos Gallego, Verónica Martínez

**Affiliations:** 1 Department of Psychology, University of Oviedo, Plaza Feijóo s/n, 33003 Oviedo, Asturias Spain; 2 Department of Experimental Psychology, Cognitive Processes and Speech Therapy, University of Complutense of Madrid, Campus de Somosaguas, Pozuelo de Alarcón, 28223 Madrid, Spain

**Keywords:** Autism spectrum disorders, Gaze following, Eye-tracking, Learning new words, Early diagnosis

## Abstract

The aim was to test the use of eye-tracking methodology for the early detection of ASD in a task of association between unfamiliar objects and pseudowords. Significant differences were found between ASD (n = 57) and TD (n = 57) Spanish speaking toddlers in the number and time of fixation. The TD children showed more and longer fixations on eyes and mouth while the ASD children attended almost exclusively to objects, making it difficult to integrate lexical and phonological information. Moreover, the TD toddlers looked at the mouth when the pseudoword was produced while the ASD toddlers did not. Gaze fixation on eyes and mouth during word learning recorded by eye-tracking may be used as a biomarker for the early diagnosis of ASD.

In the first stages of language development, the acquisition of new words takes place in communicative interaction. This demands sustained attention to diverse, relevant areas of the situation on the part of the learner (Yu & Smith, 2012). Specifically, the process of acquisition is facilitated by attention to the face of the interlocutor, eyes and mouth and the named object: the eyes to establish a link and to indicate that information is being shared, the mouth to complete and specify the phonological information of the word, and finally, the object in order to be able to associate it with the word. O’Connell et al. (2010) showed that when the speaker is a non-human, gaze-following upon hearing a new word is not sufficient to learn the referent of the word. It has been observed that joint attention to the referent predicts learning ability among typically developing children (Brooks & Meltzoff, 2005, 2008; Carpenter et al., 1998; Morales et al., 2000; Mundy et al., 2007; Tomasello, 1992) and children with ASD (Bono et al., 2004; Dawson et al., 2004). Therefore, attention plays an essential role in the process of language acquisition thus allowing the learner to concentrate on the relevant information for the acquisition of new linguistic and communicative patterns.

From a developmental perspective, the evolution of attention is a dynamic process in which changes are produced in the attention patterns of children. These are fundamental to linguistic development and range from disperse uncontrolled attention to a control which allows the fixing of attention on the eyes, and changing to the mouth and the object when the situation and the processing needs require it (Lewkowicz & Hansen-Tift, 2012; Morin-Lessard et al., 2019; Tenenbaum et al., 2015; Young et al., 2009). Evidence exists that this also facilitates the perception of speech and, in the long term, the acquisition of new words (Ellawadi & McGregor, 2016; Lu﻿sk & Mitchel, 2016; Patterson & Werke, 2003). It is therefore presumed that patterns of visual attention to eyes and mouth in situations of communicative interaction are an important factor in the processing of relevant phonological and lexical information (for an extensive review see Çetinçelik et al., 2021).

In children with Autism Spectrum Disorder (ASD), the visual attention pattern appears to be affected in early development. In order to objectively establish the nature and characteristics of the visual attention patterns in ASD children, many research studies have used eye-tracking methodology (Chita-Tegmark et al., 2015; Frazier et al., 2018; Murias et al., 2018). The use of eye-tracking in research gives a unique framework for a reliable and precise understanding of exactly what participants are paying attention to at each moment. If we assume the crucial role that gaze plays in this process (Frazier et al., 2017), then it would allow for making inferences with regard to cognitive, communicative and linguistic processing. Furthermore, a relationship has been found between measurements with eye-tracking and the clinical diagnosis of ASD with standard tests (Murias et al., 2018).

Therefore, a number of studies have repeatedly verified the presence of differences in this pattern in children at risk of manifesting ASD during their development, in comparison with children with typical development (TD). These studies show that ASD children pay less attention to the eyes of an adult in natural, face-to-face situations of interaction, such as when paying attention to static faces, faces which speak or faces which draw joint attention to objects (Chawarska et al., 2012; Fujioka et al., 2020; Gliga et al., 2012; Jones et al., 2008; Know et al., 2019; Merin et al., 2007; Nyström et al., 2019). However, the differences can be subtle, depending on the task (Wang et al., 2022). This anomalous early visual pattern is maintained throughout development and seems, to a certain extent, to allow the prediction of later manifestation of ASD (Shic et al., 2014; Wass et al., 2015). From a neuropsychological point of view, the lack of gaze exchange with the interlocutor may be explained as an attempt to reduce hyperactivation of the amygdala in accordance with what has been called the “eye avoidance hypothesis” (Stuart et al., 2022).

With regard to the pattern of attention to the mouth in ASD children, research has provided less conclusive data. In a systematic revision, Papagiannopoulou et al. (2014) concluded that the pattern of attention to the mouth could not be considered as a solid biomarker for the diagnosis of ASD. Similarly, Know et al. (2019) did not find differences in the number of times that ASD and TD children paid attention to the mouth. However, in some studies it has been observed that this attention pattern is lower in ASD children of two years of age than in TD children (Jones et al., 2008; Klin et al., 2002). On the contrary, Camero et al. (2021), in a study previous to this with a group of two-year-old children, observed that those children at risk of presenting ASD looked more at the mouth than TD children.

Research into the pattern of visual attention to eyes and mouth using eye-tracking has also been carried out in contexts of language acquisition. This is because this anomalous pattern seems to be related to a delay in language acquisition, thus allowing for the prediction of later difficulties. Young et al. (2009) observed that children with older siblings with ASD, and so with a risk of having the same syndrome, showed a greater pattern of attention to their mother’s eyes than to her mouth at the age of six months. These children also showed a slight delay in expressive language at 24 months. At the same time, a study was directed at the verification of differences in the acquisition of language in a group of ASD children at 37 months. It was observed that they had difficulties in directing visual patterns from the eyes to the mouth and also from the mouth to the eyes in verbal communicative interaction. While the TD children changed their gaze naturally, the ASD children moved their gaze from areas of the face to places which were outside the context of the scene (Hosozawa et al., 2012). It has also been observed that greater attention is paid to the mouth in a task of learning new words (naming an object) in children of 2 to 5 years of age (Tenenbaum et al., 2014). Even in younger children under the age of 25 months (Cambpell et al., 2014; Habayeb et al., 2021) it is a significant predictor of their later language development. Also, Norbury et al. (2010) confirmed that gaze patterns in children of 6 to 7 years of age with ASD, in a task consisting of learning new words, was qualitatively different in TD children. The ASD children fixed their gaze less on the face when joint attention was being paid to an object. However, they were more efficient than TD children in associating phonological forms with new examples, even though the phonological and semantic information learned during the task was not consolidated. This could be considered as evidence of qualitative difference in the learning of words. Although the initial figures for the learning of words may be similar in ASD children, the paths for obtaining this information could be qualitatively different, with the sound being potentially prioritized over semantic information and social signs (Howard et al., 2019). The differences in the visual attention pattern would also have consequences for receptive and expressive language. Chita-Tegmark et al. (2015) confirmed that at 36 months children with a high risk of presenting ASD showed less accuracy when associating words with images. By using eye-tracking, Habayeb et al. (2021) observed that in ASD toddlers between 10 and 15 months, who had not yet acquired their first words, gazes at the mouth were positively associated with expressive language.

However, despite the accumulation of results in this direction, both the heterogeneity of the behavior and characteristics of children at risk of presenting ASD, and the complexity of the intervening elements in communicative interaction in a social context as well as the different research methodologies employed, these do not specifically allow for definitive conclusions with respect to the exact nature of visual attention patterns in ASD children and its relationship with language acquisition. In any case, this different kind of attention pattern could have a negative impact on language development and communication (Hosozawa et al., 2012; Howard et al., 2019; Norbury et al., 2010).

Due to all of this, the visual attention pattern has become a phenotypical characteristic which would allow the identification of children with a risk of manifesting ASD, even before the end of their first year of life (Chawarska et al., 2013; Wagner et al., 2018). In this sense, the use of eye-tracking methodology over the last 10 years has made possible the consideration of special features, with relation to direction and length of gaze in ASD children, as signs or biomarkers in their early diagnosis. This permits detection and thus prediction before evident manifestation (Camero et al., 2021; Chita-Tegmark et al., 2015; Frazier et al., 2018; Jones & Klin, 2013; Murias et al., 2018; Papagiannopoulou et al., 2014). Therefore, eye-tracking can be considered as a non-invasive methodology which makes possible the detection of children with a high risk of manifesting ASD through an analysis of their visual attention patterns or gaze-following at an early age (before the age of 12 months).

Nevertheless, there are few studies that supply information on attention patterns in children at risk of manifesting ASD before the age of two years, using linguistic processing tasks and, in particular, language learning tasks. Furthermore, these studies are non-existent in the case of the acquisition of language in native Spanish speakers. There is the exception of a previous study in which it may be confirmed that in children at risk of ASD there exists this difference with respect to TD children of the same chronological age in the use of gaze during the processing of new words (Camero et al., 2021).

The present study again tests the use of eye-tracking methodology as a procedure for the early detection of ASD through gaze in a linguistic processing task, specifically in the learning of new words. However, in this case, it involves a wider sample of ASD children of between 6 and 24 months of age paired up with TD children according to their age of development. So, any differences found, if in fact they are found, could be attributed to a specific characteristic of ASD and not merely to a delay in development. Another aim of the study is the verification of the evolution of the gaze pattern according to age ranges, all of this with the purpose of contributing to the validation of an early diagnostic procedure through eye-tracking of ASD children. The benefits of an early diagnosis of the ASD condition are clear since this would allow early intervention to be carried out. It also leads to the setting up of more effective treatments and the improvement of the quality of life of the affected families and children, as well as contributing to its prevention, thus making possible appropriate social and structural changes in their environment.

## Methods

### Participants

The sample was made up of 114 Spanish toddlers. Of these, 57 participants were identified with a high likelihood of autism spectrum disorder (ASD) (12 girls and 45 boys), with a chronological age range of between 15 and 36 months (M = 27.9 and SD = 5.17); and 57 participants with typical development (TD) (26 girls and 31 boys), with a chronological age range of between 6 and 26 months (M = 14.4 and SD = 4.32). To control the age variable a sample pairing was carried out. Subjects were matched for developmental age (DA). The DA range is between 6 and 24 months, and for the 57 subjects with a high likelihood of ASD we have an M = 13.5, and an SD = 4.26, and for the 57 TD subjects an M = 13.9 and an SD = 4.24. All the participants were attending Preschool in Asturias, Spain.

The children with a high likelihood of ASD have received a first diagnosis of autism in Neuropediatric Services, Mental Health Services or Early Care Units, and they have been referred to the specific autism treatment unit “ADANSI” (Association of people with autism “Children of Silence”). The criteria for selection of the children with a high likelihood of ASD included children aged between 15 and 36 months with diagnostic reports of autism, as well as the following: significant language delay, scarce visual contact, lack of response when called by name, without hearing or vision problems, low communicative intention and scarcity or lack of capacity to imitate.

The TD sample was taken from the first year of Preschool at an Early Education School in the same location. More than 200 children were evaluated in order to select the 57 pairs based on the development age of the ASD children. Inclusion criteria for the comparison group, in the case of children between 12 and 26 months, were to score at least below 10 in the ADOS-2 schedule and below 2 in the M-CHAT test. Inclusion criteria in the sample of babies (6 to 12 months) was that they had a developmental age equal to or greater than their chronological age, with the absence of any neurological, social, intellectual, sensorial, or motor disorder as having no first-degree relatives with a previous ASD diagnosis. At the beginning of the school year, the center sent an informative letter to all the families of the children in the course for 0–3 years of age. All parents or legal guardians gave their consent to participation in the study.

### Instruments

Furthermore, a protocol of previous evaluation was applied to the entire sample to confirm inclusion in the ASD group. This consisted of three tests: The Revised M-CHAT test (M-CHAT-R/F) for the detection of autism in small children with a follow-up interview (Robins et al., 2009), the Brunet–Lezine Scale (PY.BL.R) of psychomotor development in early infancy (Josse & Pereda, 1997), and the Autism Diagnostic Observation Schedule-2 (ADOS-2)—Toddler Module and Module 1 (Spanish version) (Lord et al., 2019). The M-CHAT was answered by the caregivers at home before the interview, while ADOS-2 was carried out by one of the authors who has wide experience with this scale. The entire sample of ASD children met the established diagnostic criteria.

Table 1 shows the scores of the ASD group and the TD group on the three scales that make up the evaluation protocol for the confirmation of the diagnosis. The sample was divided into three groups according to development age. The age range of the first group was from 6 to 11 months, the second group was from 12 to 17 months, and the third group was from 18 to 24 months. Both the chronological age of the participants and the global development age on the Brunette–Lezine Scale is in months and all the participants with a high likelihood of ASD showed a global age below that of their chronological age. Furthermore, these showed a score for the diagnosis of autism of between 7 and 10 (CSS) in ADOS-2, which indicates a high likelihood of ASD, while the TD participants showed a score of between 0 and 5 (CSS). Finally, in the M-CHAT test, the total scores of the ASD group ranged between 9 and 20, which indicates a high possibility of ASD. This ranged from 0 to 2 in the TD group. This selection established very significant differences between the ASD and TD groups in the ADOS diagnostic tests (*Z* = − 9.56; *p* < .001; Cliff’s δ = 1) and M-CHAT (*Z* = − 10.01; *p* < .001; Cliff’s δ = 1).

**Table 1:** Mean, standard deviations and range of age in the three development age groups of ASD and TD children in the three diagnostic tests

Group	Age of development groups (AD)	n	Gender	Brunette–Lezine		Chronological age (CA)		ADOS (CSS)	M-CHAT
			F/M	M(SD)	Range	M(SD)	Range	Range	Range
ASD	1	17	3/14	9.29 (1.531)	6–11	25.82 (5.077)	18–35	8–10	18–20
	2	25	4/21	12.56 (1.609)	12–17	27.00 (5.135)	21–34	8–10	15–20
	3	15	5/10	19.73 (1.907)	18–24	31.73 (3.172)	25–36	7–10	12–20
TD	1	17	8/9	9.35 (1.497)	6–11	10.00 (2.263)	6–16	0–5	0–2
	2	25	9/16	13.56 (1.609)	12–17	14.20 (2.081)	11–19	0–4	0–1
	3	15	9/6	19.73 (1.907)	18–24	19.86 (2.386)	10–26	0–4	0–2

### Procedure

Gaze following (fixing and duration of gaze) was registered during the task, after application of the tests and in the same session. The task emulated a communicative situation of language acquisition in which was observed the emission of words by a human face in association with objects. In a video projected on a screen, a real face was presented which said a pseudo-word at the same time as a drawing of a pseudo-object (non-existent invented object) appeared.

The task consisted of six trials, where the first one was for training purposes. Each of these consisted of a video that began with a blue screen, a neutral color that does not influence the child’s pupil dilation, and a fixation point to direct the child’s attention to the center of the screen. This point, which was maintained for two seconds, corresponds to the baseline of the task. Next, a pseudo-object appeared and emitted an attention-getting sound while remaining in the center of the screen. When the object remained still, a female face appeared which asked the question: “What is that?” with happy and surprised intonation. At the end of each trial, the researcher pressed the key.

The face was the only visible part of the body. Immediately following this, the face said the name of the pseudo-object (a pseudo-word) with adult-directed natural speech. After hearing the pseudo-word, the image of the pseudo-object remained on the screen for two seconds. This was supposed to be the fading and processing time of the pseudo-word. After this, the image of the pseudo-object disappeared and only the face remained, saying “It’s gone! And what is it called?” The face was maintained for another two seconds.

**Fig. 1:**
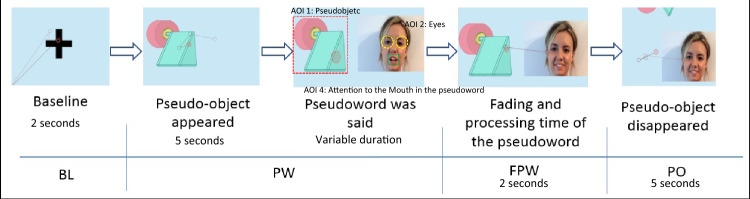
Sequence of a trial with identification of AOIs

Figure 1 represents the sequence of one of the trials. The first moment of the sequence corresponded to the baseline (BL) register (2 s); the second (2 s) and third ones corresponded to the moment of presentation of the pseudo-word (PW) (variable duration); the fourth moment was the period of time in the fading of the pseudo-word (FPW) (2 s) and the last sequence of the video was when the pseudo-object first, and later the human face, disappeared from the screen (PO) (5 s). The AOIs are also marked in the Fig. 1.

To record information, an eye-tracker apparatus, Tobii Spectrum 600 Hz, was used. The participants sat in the laps of their parents in front of a 16” monitor with a panoramic aspect ratio of 16.9 in a dark soundproof room. Their central vision was lined up with the center of the monitor, at 60 cm between the eye and the monitor. Once the participant was in place, a calibration of 5 points was carried out through colorful and attractive cartoons. This way, luminosity was controlled to ensure that changes in pupil dilatation were due to the task itself and not due to changes in the light. Before starting the task, the parents were notified of the procedure. The parent had their eyes and ears covered. The children were informed that they were going to watch TV and no further instructions were given.

A group of six pseudo-words was selected from a list of test items MEMOFON (Mariscal & Gallego, 2013). Of these, one was for training purposes (the pseudo-word *sel*). The pseudo-words were selected for being the most striking and easiest for children to attend to in a previous study (Camero et al., 2021). Also, the sample was made up of young babies, so shorter words were used to ensure attention throughout the task.

Therefore, two monosyllabic pseudo-words were selected, both with a consonant + vowel + consonant pattern (CVC) (*sel* and *muz*). *Sel* was the training pseudo-word. Three pseudo-words with two syllables were selected, a simpler one (*sina*) (CV + CV), another two were more complex since they contained an inverse syllable in a different position (*pamul* and *norba*) (CV + CVC and CVC + CV). Another pseudo-word of three syllables was also selected, with a simple pattern (*gapata*) (CV + CV + CV). Each pseudo-word was presented in association with a drawing of the pseudo-object. The pseudo-objects were designed specifically for the experiment and were randomly associated with the pseudo-words. Table 2 shows each pseudo-object associated with each pseudo-word. Once the pseudo-words were associated with the pseudo-objects, the task stimuli were shown to the different participants in random order.

**Table 2:** Pseudo-objects associated with a corresponding pseudo-word

Pseudo-osbjects	Pseudo-words	Pseudo-objects	Pseudo-words
	SEL *(trial)*		NORBA
	GAPATA		SINA
	MUZ		PAMUL

The total duration of the task was set at 10 min, although some of the participants took longer to complete it (M = 10.54; SD = 0.91; Max = 12.90). The task did not begin until the participants had become familiar with the researcher and the situation through prior play and behavior control with rewards.

During the entire task, the gaze following of the participants was recorded. Data were obtained through the system’s software “Tobii Pro Lab” and included the number and time of gazes at the previously-defined areas of interest (AOI). There were three AOIs: the woman’s mouth, the woman’s eyes, and the pseudo-object. The variables studied here were time (measured in seconds) and number of fixations on the different AOIs. Gaze fixation was considered to occur when the child’s attention was centered on the area defined for the AOI for at least 60 ms. In other cases, it was not considered that there was relevant attention. The area for each AOI was previously established by drawing it on the device. The period of time between the beginning and the end of each fixation on the AOI was considered as the fixation time. A fixation was considered to be finished when there was a shift in gaze. All of this was predefined in the Tobi Pro Lab.

Additionally, a variable of attention to the pseudo-word was included. It was a binary variable (yes or no), and it measured whether the subject was fixing their attention on the mouth when the pseudo-word was pronounced after having fixated on the eyes. In this way, we observed whether the subjects attended to the relevant phonological information.

### Data Analysis

The data obtained were analyzed with the program IBM SPSS Statistics – version 22.0 for Windows. The indices for asymmetry and kurtosis were done and a descriptive analysis of the dependent variables (number and fixation time on the different AOIs), as well as of the chronological age (CA) variable, were carried out.

Due to the violation of normality and homogeneity variance assumptions for the ASD group in some variables, the data were analyzed using nonparametric statistics.

In order to confirm whether differences existed between both groups in the gaze following measurement, pairwise Mann–Whitney U tests were used for between-group comparisons. Cliff’s Delta (δ) statistic was chosen as the effect size estimator because it is more appropriate when the homogeneity of variance or normality assumptions are violated. Based on Cohen norms, we consider an effect size of 0.2 as a small effect, 0.5 as a medium effect, and 0.8 and upwards as a large effect (Cohen, 1988). These analyses were carried out based on the total number of gaze fixations and the total time of fixations of the participants when looking at the object, the mouth, and the eyes. Friedman tests were also carried out to establish within-group differences between AOIs, and Wilcoxon signed-ranks tests were used for pairwise post hoc comparisons. Bonferroni corrections were applied to adjust the p-values for multiple post hoc comparisons.

## Results

All of the skewness and kurtosis indexes indicated symmetrical and normal distribution in the TD sample and in the global sample as well. The distribution was normal in the total number of object fixations (NOF) and object fixation time (OFT) as in the total number of fixations (TNF) and total fixation time (TFT) in the ASD group. Nevertheless, the skewness index (A) in the total number of mouth fixations (NMF), eye fixations (NEF), mouth fixation time (MFT) and eye fixation time (EFT), indicated that the distribution data was asymmetrical in the ASD group (NMF = 2.227; NEF = 2.724; MFT = 2.180; EFT = 3.903). In addition, the kurtosis index (K) indicated non-normal distribution in the same variables in the ASD group (NMF = 6.037; NEF = 7.750; MFT = 4.453; EFT = 17.323).

As expected, there were statistically significant differences between groups with regard to chronological age (*U* = 90; *Z* = − 8.709; p < .001; Cliff’s δ = 0.94), which was an average of 13.5 months older in the ASD group. The two groups were equal in development age given that they were paired according to this variable. Nevertheless, there were significant differences between groups with regard to gender, due to the greater proportion of girls in the TD group (χ^2^ = 7.73; p < .005). Subsequently, it was found that the gender variables had no significant effect on the differences found between the groups in the number of fixations and the time of fixation in the different AOIs.

### Differences Between Groups

The results showed a different attention pattern for both groups in the learning of new words. ASD children took significantly longer to complete the task than the TD children (*U* = 1187; *Z* = − 3.09; *p* = .002; Cliff’s δ = 0.27), and they gazed fewer times and for less time at the AOIs. Of the total time taken to complete the task, the ASD group used 6.76% in looking at AOIs and the TD group 10.83% (*U* = 325.5; *Z* = − 7.36; *p* < .000; Cliff’s δ = 0.80). There were statistically significant differences between the ASD group and the TD group in the total number of gaze fixations (*U* = 633; Z = − 5.62; *p* = .000; Cliff’s δ = 0.61) and the total time of fixations (*U* = 368.5; *Z*= − 7.12; *p* < .000; Cliff’s δ = 0.77). Specifically, ASD children gazed at the eyes and at the mouth fewer times (*U* = 267.5; *Z*= − 7.74; *p* < .001; Cliff’s δ = 0.84; *U* = 546.5; *Z*= − 6.11; *p* = .000; Cliff’s δ = 0.66) and for less time than the TD children (*U* = 285; *Z*= − 7.64; *p* < .001; Cliff’s δ = 0.83; *U* = 659; *Z*= − 5.47; *p* = .000; Cliff’s δ = 0.59). However, the ASD children gazed more at the objects (*U* = 1060; *Z*= − 3.20; *p* < .001; Cliff’s δ = 0.35) and for a longer time (*U* = 1120; *Z*= − 2.86; *p* < .004; Cliff’s δ = 0.31). Moreover, they almost never gazed at the mouth when the pseudo-word was emitted. As shown in Table 3, the performance of the groups in number and time of gaze fixation showed significant differences in all AOIs.

**Table 3:** Median, IQR, differences between groups, *p* values, and effect size in number of fixations and gaze fixation time on the areas of interest between ASD and TD.

		ASD		TD				
		MDN	IQR	MDN	IQR	*Z*	*p*	Cliff’s δ
Eyes	NEF	2	6.5	37	24	− 7.74	0.000	0.84
	EFT	0.44	1.99	23.45	19.12	− 7.64	0.000	0.83
Mouth	NMF	4	16	33	32.5	− 6.11	0.000	0.66
	MFT	2.50	8.1	19.16	23.34	− 5.47	0.000	0.59
Object	NOF	77	34.5	57	33	− 3.20	0.000	0.35
	OFT	33.08	11.95	27.6	14.23	− 2.86	0.004	0.31

Given that the time taken to complete the task was not the same for both groups, analyses were carried out with proportional measurements, taking the percentage of the time that the participants spent looking at the different AOIs in relation to the total time used in completing the task.

Table 4 shows the percentages of fixation time on each AOI by group in relation to the total time taken on the task. It also shows the differences between groups and the *p* values. As can be observed, significant differences exist in the percentage of time spent looking at the eyes and at the mouth (p < .001), which is greater for the TD children, and in the time spent looking at the objects (p = .034), which is greater for the ASD children.

**Table 4:** Median, IQR, differences between groups, *p* values, and effect size in percentage of fixation time on the areas of interest between ASD and TD.

	ASD		TD		*Z*	*p*	Cliff’s δ
	MDN	IQR	MDN	IQR			
% EFT	3.89	3.19	0.07	0.32	− 7.802	< 0.001	0.84
% MFT	3.04	3.86	0.42	1.25	− 5.62	< 0.001	0.61
% OFT	4.40	2.13	4.91	2.11	− 21.02	0.034	0.23

*EFT* eyes fixation time, *MFT* mouth fixation time, *OFT* object fixation time.

**Fig. 2:**
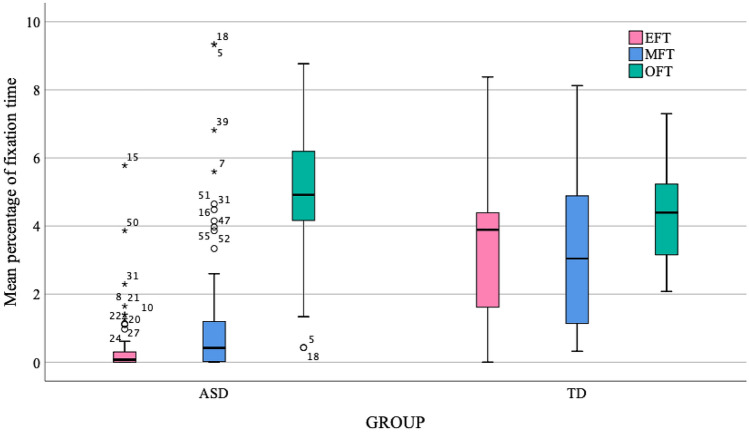
Mean percentage of fixation time to AOIs in ASD and TD

Figure 2 shows the mean percentages of time of fixation on the eyes, mouth and object for both groups. Also, a significant number of outliers can be observed in the ASD group.

It should be pointed out that a large number of ASD children never looked at the eyes (25 of them, 43.86%), while in the TD group only one child did not do this. A considerable percentage of ASD children did not look at the mouth (11 of them, 19.30%). All of the TD children fixed their gaze on the mouth and all of the children, both TD and ASD, made more fixations on the objects. If the average time for each fixation is considered, the TD children only made significantly longer fixations than the ASD children when looking at the eyes (U = 0.515; Z= − 3.301; *p* < .001). However, the differences between TD and ASD children are not significant in the average time dedicated to each fixation on the mouth (*p* = .143) and object (*p* = .318).

In accordance with our data, the distribution of the participants showed that, with respect to the pattern of joint attention and attention to the mouth during the production of pseudo-words, only two participants from the ASD group were unable to be differentially classified from the participants of the TD group. This means that the procedure had detected the ASD participants in 96.5% of cases. It may be inferred from this that the procedure is effective for the detection of ASD.

This study also registered whether the children looked at the mouth when pseudo-words were emitted. Of the 57 TD children, all of them looked at the mouth two or more times when the pseudo-word was emitted: 78.9% (45) did this during the five tests, 12.3% (7) did this in four of the tests, 5.3% (3) in three of the tests, and 3.5% (2) in two of the tests. In contrast, in the ASD group, 53 of the 57 participants (92.98%) never paid attention to the mouth during the production of the pseudo-word: one participant did this in all five tests (1.75%), two participants in two tests (3.5%) and one participant in only one test (1.75%).

### Within-Group Differences

With respect to the specific performance of each group during the task, the within-group differences were statistically significant in the number of fixations in the different areas in the TD group (χ^2^ = 31.09; *p* < .001) and also in the ASD group (χ^2^ = 85.87; *p* < .001). Pairwise post hoc comparisons showed that both groups presented a pattern with a predominance of fixations on the objects. They made more fixations on the object rather than on the eyes, and on the object rather than the mouth. In neither of the groups the difference between fixations on eyes or mouth was significant, although in the TD children the difference was close to significance in favor of the eyes. As for the ASD children, these differences between fixations on eyes and mouth were not significant due to their scarce number in both areas, although the number of fixations on the mouth was slightly greater than on the eyes.

Within-group comparisons also showed statistically significant differences in the time of gaze fixation in the same direction as in the number of fixations, TD group (χ^2^ = 12.35; *p* < .002) and ASD group (χ^2^ = 79.29; *p* < .001). Pairwise post hoc comparisons showed TD children spent more time looking at the object rather than at the eyes, and at the object rather than at the mouth. However, there were no significant differences between the time of gaze fixation on the eyes and on the mouth. ASD children spent more time looking at the object rather than at the eyes and at the object rather than at the mouth, as well as at the mouth rather than at the eyes in this group. In this case, all the differences were statistically significant (Table 5).

**Table 5:** Differences Within-group, *p* values, and effect size in number of fixations and gaze fixation time on the areas of interest between ASD and TD.

	ASD			TD			
	*Z*	*p*	Cliff’s δ	*Z*	*p*		Cliff’s δ
NEF- NMF	− 2.70	0.07	0.31	− 0.63	0.53		0.05
NEF-NOF	− 6.57	0.000	0.97	− 4.92	0.000		0.57
NMF-NOF	− 6.51	0.000	0.91	− 5.58	0.000		0.62
EFT-MFT	− 2.70	0.007	0.31	− 2.78	0.781		0.03
EFT-OFT	− 6.48	0.000	0.94	− 2.63	0.008		0.33
MFT-OFT	− 6.51	0.000	0.80	− 3.80	0.000		0.38

*NEF* number of eye fixations, *EFT* eyes fixation time, *NMF* number of mouth fixations, *MFT* mouth fixation time, *NOF* Number of object fixations, *OFT* object fixation time

### Evolution by Age Group

**Fig. 3:**
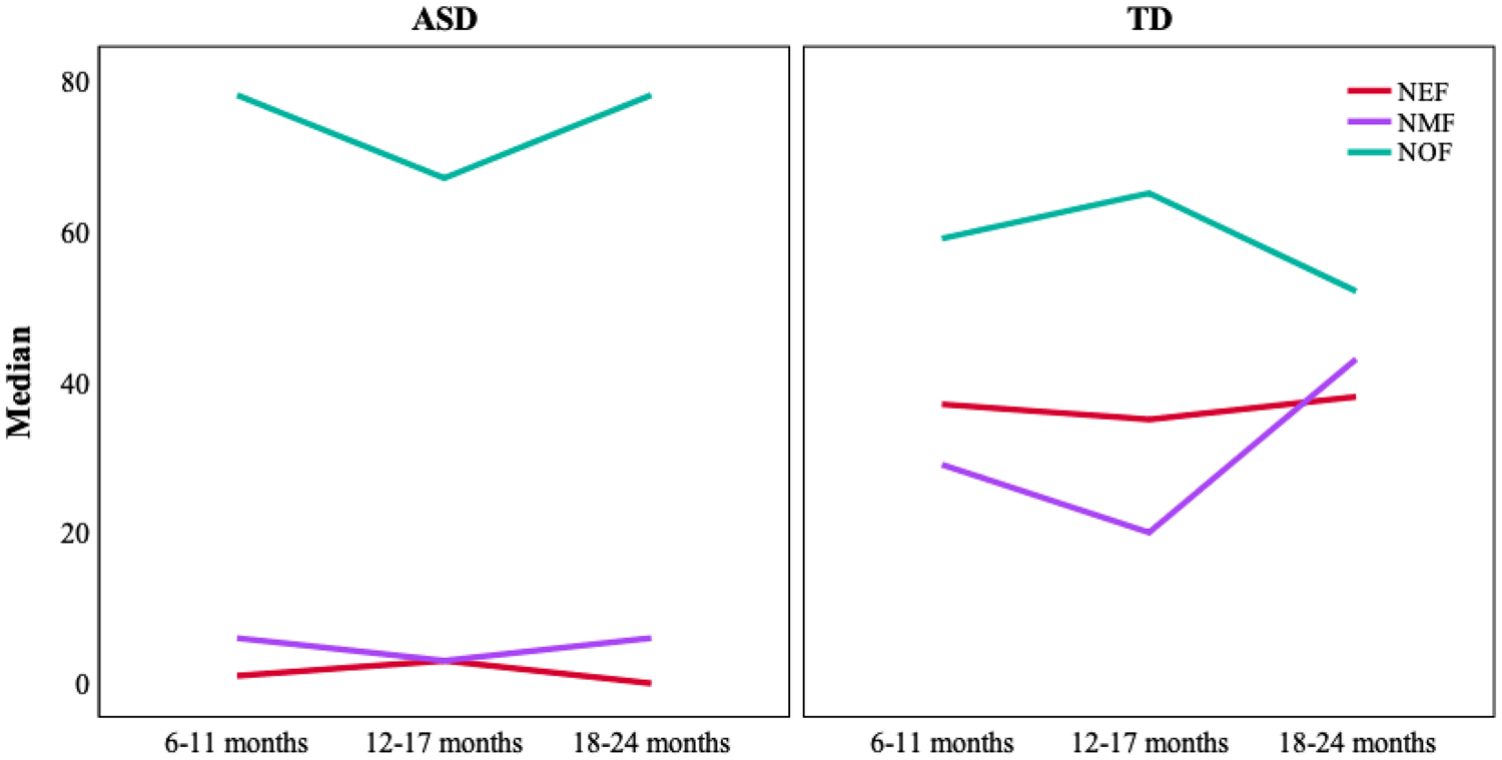
Evolution of the number of gaze fixations by age group in the AIOs in ASD and TD children

**Fig. 4:**
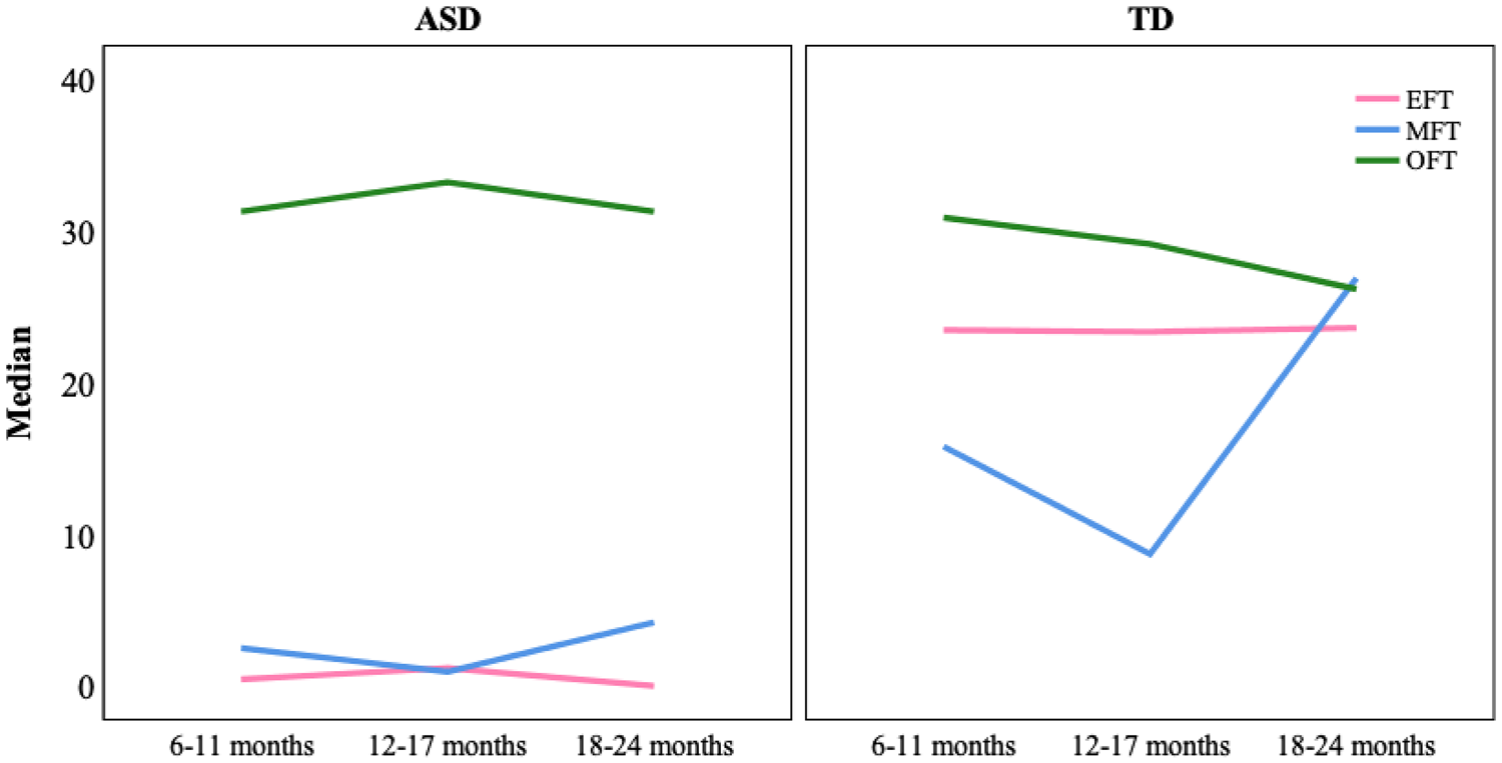
Evolution of the number of gaze fixations by age group in the AIOs in ASD and TD children

Finally, with regard to the evolution by age group within each group of participants (ASD and TD), an evolutionary pattern of change was not detected in either the number of gaze fixations or in the time of gaze fixations (Figs. 3 and 4). There were no statistically significant differences between the groups of children at a younger development age and the groups of children with an older development age.

The Kruskal–Wallis test showed the existence of significative differences in number and time of gaze fixation in all AOIs (p < .000 to eyes and mouth; p < .01 to number of object fixations) except for object fixation time which was close to significance (p = .066). Comparisons by pairs showed significant differences between TD and ASD children in all age groups, except for NOF and OFT in which the differences were only significant for the groups of 18 to 24 months. Even in the number and time of gaze fixations on the eyes, the differences were significant between the youngest TD children (6–11 months) and the older ASD children (18–24 months). The fact that they had produced significant differences in NOF and OFT at 18–24 months, which had not appeared in younger children, was due to a decrease of NOF and OFT in the TD group in this age range (Table 6).

**Table 6:** Median, IQR, differences between groups, *p* values, and effect size in number of fixations and gaze fixation time on the areas of interest between ASD and TD children by age group

			ASD		TD				
			MDN	IQR	MDN	IQR	*Z*	*p*	Cliff’s δ
6–11 m		NEF	1	7	37	41	− 4.17	.000	.83
		NMF	6	11	22	27	− 2.91	.003	.74
		NOF	78	49	59	31	− 1.57	.122	.32
		EFT	0.44	1.69	23.45	30.2	− 3.93	.000	.79
		MFT	2.50	6.88	12.81	18.7	− 2.46	.013	.55
		OFT	31.28	13.47	30.88	11.64	− 1.02	.322	.20
12–17 m		NEF	3	11	35	26	− 4.74	.000	.78
		NMF	3	12	15	34	− 2.86	.004	.63
		NOF	67	33	65	34	− 1.14	.256	.15
		EFT	1.18	5.7	23.34	20.19	− 4.87	.000	.80
		MFT	0.94	7.13	5.53	22.7	− 2.69	.007	.62
		OFT	33.22	15.06	29.14	11.5	− 1.50	.133	.25
18–24 m		NEF	0.00	2	38	14	− 4.19	.000	.88
		NMF	6	27	36	18	− 2.61	.008	.72
		NOF	78	33	52	36	− 2.95	.002	.63
		EFT	0.00	0.59	23.6	11.3	− 4.18	.000	.88
		MFT	4.2	19.92	21.88	15.73	− 2.51	.011	.69
		OFT	31.28	15.84	26.16	13.24	− 2.39	.016	.51

## Discussion

The main objective of this study was to test the use of eye-tracking methodology as a procedure for the early detection of ASD through gaze-following in a linguistic processing task, specifically the learning of new words by native Spanish speakers. In a previous study, Camero et al. (2021) showed the validity of this procedure to distinguish between TD children and children at risk of ASD with the same chronological age. In the present study, the results have been obtained by comparing attentional behavior, measured by registering gaze fixation and time of gaze with TD children and older children at risk of ASD. These were paired up according to development age. Thus, the differences obtained may not be attributed to general development, but rather to specific characteristics of the ASD phenotype.

In this study it has been shown that the attention to relevant areas of a scene (AOIs) of children at risk of manifesting ASD, while listening to new words associated with the presence of objects, is different to that of TD children at the same level of development. This is in accordance with what is already known about attention in this population (see Frazier et al., 2017). They present a lower number of fixations on the AOIs of the interlocutor (eyes and mouth) and a lower time of attention to these. In general, the children at risk of manifesting ASD present 30% less attention to all the AOIs taken together. Also, this attention is directed with a marked preference at the objects. They direct more attention to the objects than TD children. The total time of attention directed at the eyes and mouth is 85% lower than that of TD children.

The greatest difference between participants at risk of ASD and TD children is that ASD children look significantly less and for less time at the eyes than do TD children. This is a robust effect which corroborates once again the well-established and well-known fact of reduced eye contact with the interlocutor of ASD children. This starts from the age of six months in situations of natural face-to-face interaction, of joint attention or attention to a static or dynamic face (Chawarska et al., 2012; Jones et al., 2008; Know et al., 2019; Merin et al., 2007; Nyström et al., 2019; Shic et al., 2014; Wang et al., 2022; Wass et al., 2015).

Similarly, children at risk of manifesting ASD also looked less and for less time at the mouth during the task than TD children and especially, they did not look at the mouth during the emission of the pseudo-word. In this case, although the size of the effect did not reach the dimension of that found for eye contact, it was also well-established. Therefore, this corroborates the fact that ASD children do not pay the necessary attention to the movements of the mouth, and this may even be non-existent when listening to new words. While carrying out the task, it was observed that when the TD children looked at the mouth when the pseudo-word was pronounced, which they did on practically every occasion, their gaze always proceeded from the eyes. This was a constant pattern that was not observed in the ASD children. This attention pattern of a shift from eyes to mouth coinciding with the emission of the pseudo-word would supposedly allow a better phonological identification of new words and so, of more fluid learning and better-quality learning. Lack of attention to the mouth is in keeping with the results obtained in other research (Jones et al., 2008; Klin et al., 2002). However, there is no clear consensus in the scientific literature about whether ASD children pay less attention to the mouth as a phenotypical feature of their condition. In a previous study using the same task of learning of words, although with a much smaller sample (Camero et al., 2021), this was not confirmed, even though ASD children were compared with TD children of the same chronological age, and so, with greater cognitive development. The lack of clear results in the literature calls into question whether the pattern of eye fixation on the mouth could be used as a biomarker for the diagnosis of ASD or at least as an indicator of the presence of an ASD condition (Know et al., 2019; Papagiannopoulou et al., 2014). Differences in the consideration of the pattern of eye fixation on the mouth as a biomarker could be due to the type of task used. While in Know et al. (2019) the participants saw comics in which a face appeared at the same time as the person made gestures with their hands and produced a sentence, our study deals with a task of pure learning of words in which attention to the mouth would be relevant for the identification of phonemes. Thus, this would not only be part of social contact but also a useful resource for the identification of the constituent phonemes of new words. In this sense, Norbury et al. (2010) confirmed in an analogical task that the greater number of fixations on the mouth in ASD children could be associated with a greater communicative and linguistic competence. Similarly, the number of fixations on the mouth has been considered as a significant predictor for later linguistic development between the ages of 10–15 months and five years (Campbell et al., 2014; Habayeb et al., 2021; Tenenbaum et al., 2014; Young et al., 2009). In the present study, the children at risk of manifesting ASD hardly ever paid attention to the mouth at the moment in which the pseudo-word was produced, while 80% of the TD children did this on every occasion and 90% did this on more than 80% of occasions. This different pattern could explain so many later difficulties in elaborating consistent phonological and lexical representations in ASD children (Chita-Tegmark et al., 2015). In sum, this would affect the learning of words and the development of language. Therefore, attention to the mouth could also be essential in the same way that eye contact is essential for labelling an object with a certain word (Nystrom et al., 2019), as it seems to play a critical role in visual speech segmentation (Lusk & Mitchel, 2016). It has been confirmed that, in communicative verbal interaction, children who present ASD have difficulties in directing their visual pattern from the eyes to the mouth, as well as from the mouth to the eyes (Hosozawa et al., 2012).

Even so, and although attention to the mouth is very scarce, the ASD children paid more attention to the mouth than to the eyes, though the difference was small and non-significant. This was due to an almost complete lack of attention paid to the eyes. On the other hand, the TD children paid more attention to the eyes than to the mouth in absolute terms, but the difference was also non-significant. The scarcity of gaze and time of attention dedicated to the eyes and mouth manifested by the children at risk of presenting ASD contrasts with the fact that when they looked at one of the AOIs, they spent most of that time looking at the objects than at anything else. The ASD children paid attention almost solely to the object. They directed 80% of their gazes and 74% of attention time to fixation on the objects. This preference for objects during the word-learning task is one more manifestation of the general preference for objects of the ASD children in any situation. However, in this case, it can be observed that even with verbal stimuli that require attention, the preference for objects is maintained. The TD children also looked at the objects more than at the eyes and mouth, but they did this in quite a lower proportion, 55% and 49% respectively. Therefore, the percentage of time that the participants centered their attention on the object in comparison with eyes and mouth was much greater in ASD children than in TD children. Even though the TD children also looked more at the objects than at the eyes and mouth, they did not do this in such an unbalanced way, since they divided their attention among the three elements. In fact, in the case of the objects, the differences between ASD and TD children went in the opposite direction of that confirmed for eyes and mouth. The ASD children looked more times and for longer times at the objects than the TD children, although the size of the effect was small. To correctly interpret the fact that both groups look more and for longer times at the objects than at any other AOI, it must be taken into account that these are new and attractive objects to which attention must be paid in order to correctly learn their association with a word.

Therefore, the TD children with an age of development of between 6 and 24 months in a communicative situation in which new objects are associated with new words divide their attention among the three elements involved. They pay attention to the face of the interlocutor, eyes, and mouth, as well as to the object. This attention pattern makes possible the recognition of the object, the perception of the relationship and the communicative intention of the interlocutor as well as the identification of the surface orofacial movements that accompany the words. Thus, they associate the object and the word and create a lexical label for the object (Çetinçelik et al., 2021; Patterson & Werke, 2003). In contrast, the children at risk of presenting ASD fix their attention on the object, thus missing the visual information associated with the articulation of the word. This, along with the lack of perception of the communicative intention of the speaker, impedes establishing a relationship between the word and the object. So, this poses difficulties in the acquisition of new lexical labels. This different attention pattern could contribute to an explanation of why the language of ASD children follows different qualitative paths to those of TD children (Howard et al., 2019; Norbury et al., 2010).

From a developmental point of view, these differences in looking at the eyes and mouth between ASD and TD children seem to remain constant between the ages of 6 to 24 months, except for the objects in which differences do not appear until 18 months. Even when comparing the youngest TD children (6–11 months) and the oldest ASD children (18–24 months) differences are found in favor of the former in looking at eyes and mouth. With regard to the objects, between 18 and 24 months the differences that appear are due to a decrease in the number and time of fixations on objects by TD children. On the other hand, there does not appear to be a significant developmental change in each of the TD and ASD groups between the ages of 6 and 24 months. The scarcity of gaze at eyes and mouth and the greater attention paid to objects is maintained constant during the entire time in those children at risk of presenting ASD. Only in the older children, between 18 and 24 months, is there a slight increase in looking at the objects. Therefore, it seems that the attention pattern examined is established at a very early age and remains constant. Fujioka et al. (2020) point out that it is from three years onward when the percentage of time of fixation on the eyes increases in TD children, even though this is not so in ASD children. In the case of ASD children, the lack of development in the attention pattern could be explained by the difficulties in basic social/non-social discrimination processes (Frazier et al., 2017). In accordance with all of this, the markedly different attention pattern to eyes, mouth and object found in native Spanish-speaking ASD children of between 6 and 24 months of age, in a situation that emulates the learning of new words, could be considered as a specific characteristic of ASD and not a mere consequence of a non-specific developmental delay.

In conclusion, the use of eye-tracking technology in this task is confirmed to be an adequate and useful technique in the early diagnosis of ASD (Frazier et al., 2017; Habayeb et al., 2021; McPartland et al., 2020). This is due to the fact that it allows for precise detection and characterization of the anomalous attention pattern which is a prominent and universal characteristic of ASD children (Chawarska et al., 2013; Hosozawa et al., 2012; Jones et al., 2008; Shic et al., 2011) and a powerful biomarker for early diagnosis (Camero et al., 2021; Know et al., 2019; Papagiannopoulou et al., 2014). The fact that this has been tested using a new word-learning task increases its value, given that this task paradigmatically requires the use of a pattern of joint attention. The results obtained from our study allow to establish that the effectiveness of eye-tracking during a word-learning task in the early detection of ASD can be fixed at over 95%. Consequently, the identification of an anomalous attention pattern as a biomarker in children at risk of presenting ASD through eye-tracking in a linguistic processing task would be valid for early diagnosis of ASD (Camero et al., 2021; Chita-Tegmark et al., 2015; Frazier et al., 2018; Jones & Klin, 2013; Murias et al., 2018; Papagiannopoulou et al., 2014).

There are some limitations in this study. First, the study was not carried out in a natural face-to-face situation but rather, the children watched a video in which a real person’s face pronounced a word associated with an object. Therefore, our results may not be generalized to a natural communicative situation. Secondly, the amount of time that the children looked at other elements of the screen, different to the eyes, mouth, and objects, was not registered. Finally, we used only a TD control group matched to the ASD group in developmental age in the study. Matching in both developmental age and chronological age would have completely ruled out age effects.
